# DPTG: diffusion policy with tactile feasibility guidance

**DOI:** 10.3389/frobt.2026.1851102

**Published:** 2026-06-10

**Authors:** Jin Liu, Hongzhan Yu, Can Zhao, Haonan Zhao, Zhemeng Zhang, Daolin Ma, Weiming Wang

**Affiliations:** 1 School of Mechanical Engineering, Shanghai Jiao Tong University, Shanghai, China; 2 Xense Robotics, Shanghai, China; 3 School of Ocean and Civil Engineering, Shanghai Jiao Tong University, Shanghai, China

**Keywords:** contact-rich manipulation, diffusion policy, feasibility guidance, tactile sensing, vision-tactile manipulation

## Abstract

Robotic manipulation in contact-rich environments requires integrating vision and tactile sensing, yet effective fusion of these modalities remains challenging. Existing methods often adopt symmetric feature fusion or joint policy learning, implicitly treating tactile as a continuous motion generator comparable to vision. However, this misaligns with the nature of touch, which primarily provides physical constraints and phase cues rather than action proposals. In this work, we propose DPTG, a framework that reformulates tactile sensing as a physical feasibility constraint rather than a parallel action generator. Actions are sampled from a vision-driven diffusion policy and guided by a tactile feasibility classifier. To extract phase-relevant cues, we derive an adaptive guidance schedule from feasibility scores, selectively activating constraints only when contact is informative. Moreover, the feasibility classifier is trained as a standalone module using interaction signals, enabling classifier reuse across related tasks that share the same action space and tactile setup. Experiments in simulation and the real world demonstrate that DPTG improves success rates while reducing peak forces, leading to safer and more stable contact interactions than baselines.

## 1 Introduction

Robotic manipulation in the real world requires the integration of heterogeneous sensory modalities, among which vision and touch play fundamentally distinct roles. Vision provides global geometric and semantic information that supports long-horizon planning and object localization, while tactile sensing delivers localized, contact-specific feedback that is critical for precise physical interaction Johansson and Westling (1984); Jenmalm and Johansson (1997); Li et al. (2020). Despite recent progress, effective vision–tactile integration remains challenging, especially in contact-rich manipulation where tactile observations are temporally sparse and contact dynamics are uncertain.

Most existing visuotactile policies treat sensory modalities symmetrically, either by concatenating multimodal features or by jointly learning end-to-end action generators Hansen et al. (2022); Yuan et al. (2024); Suresh et al. (2024); Wu et al. (2025a). Such designs implicitly assume that tactile sensing should continuously contribute to action generation, in a manner comparable to vision. However, tactile feedback is inherently spatially local and becomes informative primarily during brief contact phases. In the tight-clearance settings considered here, symmetric fusion strategies yield only limited gains over vision-only policies and can exhibit less stable contact behavior, such as force spikes or jamming.

**FIGURE 1:**
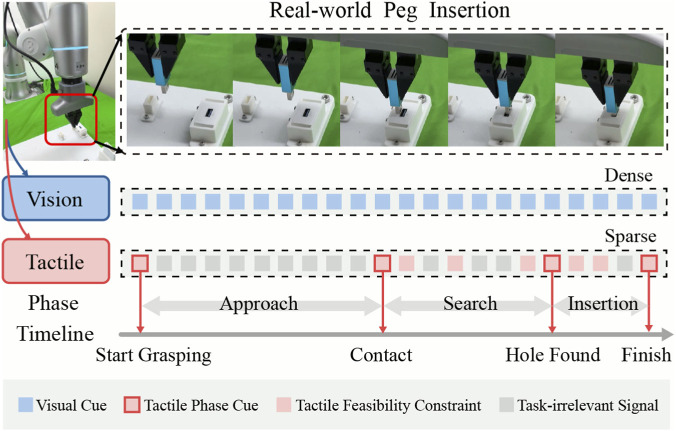
Asymmetric modalities. Vision and tactile sensing play fundamentally different roles in contact-rich manipulation. Vision provides continuous, task-level guidance for action generation, whereas tactile feedback is sparse, contributing through phase cues and feasibility constraints.

In this work, we adopt a different perspective on vision-tactile integration. Rather than modeling tactile sensing as a competing policy or a direct contributor to action generation, we argue that touch primarily provides phase awareness and feasibility constraints. Vision determines what action to take based on global geometry and task intent, while tactile feedback informs when and whether an action is physically admissible. This asymmetric formulation more faithfully reflects the physical roles of the two modalities in contact-rich manipulation, as shown in Figure 1.

Building on this insight, we propose DPTG, a diffusion-based control framework where actions are sampled from a vision-driven diffusion policy and guided by the gradient of a tactile feasibility classifier. To extract the phase-relevant cues from tactile feedback, we derive an adaptive guidance schedule directly from the feasibility score, selectively activating tactile constraints when contact becomes informative. This yields implicit phase awareness without explicit phase supervision.

Moreover, the feasibility classifier can be trained as a standalone tactile constraint module with rule-assisted labels derived from interaction signals, enabling task-agnostic reuse across downstream tasks sharing the same action space and tactile setup. Experiments in both simulation and on real robots show that DPTG improves task success under the evaluated conditions while reducing peak contact forces and force variance, resulting in more stable and safer contact interactions than the baseline methods.

Overall, the contributions of this work are:

- A feasibility-centric formulation of vision–tactile manipulation. We introduce a novel modeling perspective that reformulates tactile sensing as a source of physical feasibility and phase awareness, rather than as an action-generating modality, and instantiate this formulation via a classifier-guided diffusion policy that better matches the temporally sparse and spatially local nature of tactile feedback.
- A task-agnostic and reusable tactile constraint module. We decouple tactile modeling from task policy learning by training a standalone feasibility classifier with rule-assisted labels derived from interaction signals, enabling task-agnostic reuse across tasks sharing the same action space and tactile setup, as well as plug-in integration through gradient-based guidance.
- Improved stability and safety in contact-rich tasks. We show that incorporating tactile guidance at inference time suppresses force spikes and reduces contact-force variance, yielding more stable interactions than vision-only and symmetric visuotactile baselines.

## 2 Related work

Our work relates to recent advances in vision–tactile fusion for robotic manipulation and diffusion-based policy learning. We review these two lines of research and highlight the gaps that motivate our asymmetric, feasibility-guided formulation.

### 2.1 Vision–tactile fusion in robotic manipulation

Vision–tactile fusion has been widely explored to improve robustness in contact-rich manipulation, leveraging the complementary strengths of global visual perception and local contact feedback.

A representative line of work studies end-to-end visuotactile policy learning, where visual and tactile observations are treated as comparable inputs and jointly fused for manipulation. Early works show that tactile feedback complements vision in stabilizing grasps and predicting contact outcomes Calandra et al. (2017); Lee et al. (2020); Ding et al. (2023). Subsequent works integrate tactile and vision information into deep reinforcement learning frameworks, demonstrating improved performance in grasping, insertion, and dexterous manipulation tasks Hansen et al. (2022); Lee et al. (2024); Pattabiraman et al. (2024); Su et al. (2024); Zhao et al. (2025). While effective, these methods typically rely on one module or symmetric fusion, implicitly assuming continuous and comparable contributions from both modalities throughout execution.

Another stream of work explores intermediate visuotactile representations to align tactile sensing with visual perception geometrically or structurally. Representative examples include Robot Synesthesia and 3D-ViTac, which embed tactile feedback into shared 3D or point-based representations with vision Qin et al. (2023); Yuan et al. (2024); Huang et al. (2025), as well as NeuralFeels, which models visuotactile perception as a continuous neural field Suresh et al. (2024). More recently, self-supervised multimodal representation learning has emerged as a scalable paradigm for visuotactile fusion. Contrastive pretraining, masked multimodal learning, and unified tactile representations enable generalizable visuotactile features across sensors, tasks, and embodiments Dave et al. (2024); Sferrazza et al. (2024); Xu et al. (2024). Several works further leverage large-scale human or robotic interaction data to learn transferable vision-touch representations without task-specific supervision Gu et al. (2025); Liu et al. (2025); Jiang et al. (2025); Wu et al. (2025a).

Despite these successes, most existing approaches treat vision and touch as comparable information sources for continuous action generation. This overlooks the inherently sparse, local, and phase-dependent nature of tactile feedback, which primarily provides feasibility constraints and contact state cues during interaction rather than continuous action proposals. Closest to our work, Chen et al. (2025) improves robustness by combining modality-specific policies, but does not explicitly model the feasibility-oriented and phase-dependent role of tactile sensing during contact.

### 2.2 Diffusion policies for robotic control

Diffusion models have recently emerged as a powerful framework for robotic policy learning, enabling expressive action distributions and stable training through iterative denoising Wolf et al. (2025). Diffusion Policy formulates visuomotor control as conditional action diffusion and demonstrates strong performance across a wide range of manipulation tasks Chi et al. (2023). Building on this foundation, subsequent work explores improved conditioning and representations for diffusion-based control. DP3 incorporates compact 3D scene representations into diffusion policies to improve generalization across viewpoints and tasks Ze et al. (2024). In contact-rich settings, diffusion policies have been extended to incorporate tactile or force feedback for reactive control.

Reactive Diffusion Policy introduces a slow–fast hierarchy that combines low-frequency diffusion planning with high-frequency tactile or force corrections Xue et al. (2025). Tactile-specific diffusion formulations further explore modeling actions directly in force or contact-related spaces, demonstrating improved precision in insertion and force-aware manipulation tasks Wu et al. (2025b); Helmut et al. (2025). In parallel, diffusion-based planning and guidance frameworks such as Diffuser illustrate how constraints or rewards can guide denoising trajectories to shape behavior Janner et al. (2022), and recent surveys summarize the growing role of diffusion models in robotic manipulation Wolf et al. (2025).

While recent diffusion-based policies incorporate tactile or force information, most integrate tactile sensing through direct conditioning or joint multimodal training, tightly coupling action generation with tactile modeling. Consequently, they do not explicitly separate task-level action generation from contact feasibility reasoning, nor provide a principled, phase-aware mechanism for constraint-driven reshaping of the action distribution during inference.

## 3 Methods

### 3.1 Problem formulation

We consider visuotactile manipulation with multimodal observation $o_{t}=\left\{o_{t}^{v},o_{t}^{h}\right\}$ , where $o_{t}^{v}$ denotes visual observations and $o_{t}^{h}$ denotes tactile observations. The robot executes continuous actions $a\in R^{d}$ (or short action chunks). Our goal is to learn a policy $\pi \left(a|o_{t}\right)$ that exploits the asymmetric roles of the two modalities: *vision proposes task-relevant actions*, while *touch provides feasibility constraints and phase awareness*. We adopt a diffusion-based action generator driven primarily by vision and incorporate tactile information through a feasibility classifier that constrains the sampling process. In our implementation, a denotes a normalized short-horizon chunk of end-effector Cartesian pose increments; it is denormalized, clipped, and sent to the robot control stack before execution.

### 3.2 DPTG: diffusion policy with tactile guidance

We model action generation using a classifier-guided diffusion policy. Let $a_{k}$ denote the action sample at diffusion step k , progressing from an initial noisy sample (k=K) to a clean action (k=0) . The reverse diffusion update used for inference is:

$$a_{k-1}=a_{k}-\eta_{k}\left(\epsilon_{\theta }\left(a_{k},o_{t}^{v},k\right)+\lambda \left(o_{t}^{h}\right)\;\nabla_{a_{k}}\log C_{\phi }\left(o_{t}^{h},a_{k}\right)\right).$$ (1)

This equation defines the complete inference algorithm. The three terms inside the parentheses play distinct roles:

-
$\epsilon_{\theta }\left(a_{k},o_{t}^{v},k\right)$ : a vision-driven diffusion term that generates task-relevant actions conditioned on global geometry and semantic context;
-
$\nabla_{a_{k}}\log C_{\phi }\left(o_{t}^{h},a_{k}\right)$ : a tactile feasibility guidance term that biases sampling away from physically infeasible actions;
-
$\lambda \left(o_{t}^{h}\right)$ : a scalar that modulates the strength of tactile guidance and enables phase-aware control.

**FIGURE 2:**
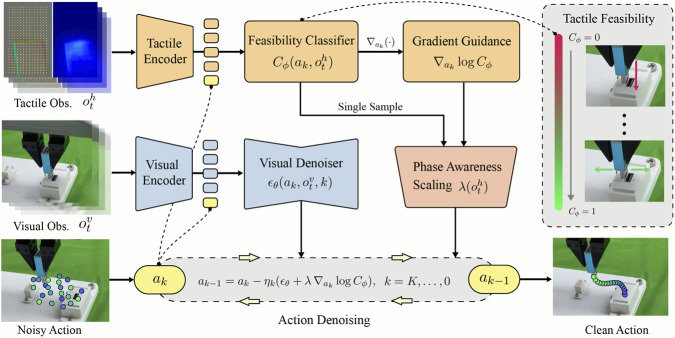
The architecture implements an asymmetric visual-tactile fusion via classifier-guided diffusion. The Visual Denoiser $\epsilon_{\theta }$ (blue block) serves as the primary policy, generating task-relevant action proposals based on global visual context $o_{t}^{v}$ . This guidance is dynamically modulated by the Phase Awareness Scaling term $\lambda \left(o_{t}^{h}\right)$ to adaptively enforce constraints during contact-rich phases, resulting in the combined update rule shown in Equation 1.

The overall framework is illustrated in Figure 2. In the following, we describe each component in detail.

### 3.3 Vision-based diffusion policy

The backbone of our framework is a vision-driven diffusion policy that serves as the sole action generator. The denoising network $\epsilon_{\theta }\left(a_{k},o_{t}^{v},k\right)$ predicts the noise added at diffusion step k conditioned only on visual observations.

The policy is trained using the standard DDPM objective Ho et al. (2020):

$$L_{\text{diff}}=E_{a_{0},\epsilon ,k}\left[{\left\|\epsilon -\epsilon_{\theta }\left(a_{k},o_{t}^{v},k\right)\right\|}_{2}^{2}\right],$$ (2)

where the noisy action $a_{k}$ is obtained by the forward diffusion process:

$$a_{k}=\sqrt{{\bar{\alpha }}_{k}}\;a_{0}+\sqrt{1-{\bar{\alpha }}_{k}}\;\epsilon ,\;\epsilon ∼N\left(0,I\right).$$ (3)

This vision-centric policy captures global geometry and task semantics, and provides the proposal term used in Equation 1. Tactile observations are intentionally excluded from action proposal to avoid overfitting to sparse and noisy contact signals.

### 3.4 Feasibility classifier as tactile constraint

To incorporate tactile information, we introduce a feasibility classifier $C_{\phi }\left(o_{t}^{h},a\right)\in \left[0,1\right]$ that estimates whether executing action a under the current tactile observation is kinematically feasible and contact-safe:

$$C_{\phi }\left(o_{t}^{h},a\right)\approx P\left(\text{feasible}|o_{t}^{h},a\right).$$ (4)

Inspired by classifier-guided diffusion Dhariwal and Nichol (2021), we use the gradient

$$\nabla_{a_{k}}\log C_{\phi }\left(o_{t}^{h},a_{k}\right)$$ (5)

to bias the diffusion sampling process away from infeasible actions. As shown in Equation 1, this guidance term acts as an independent constraint on candidate actions proposed by the vision diffusion policy.

Unlike standard classifier guidance, $C_{\phi }$ does not encode a target action distribution or task intent; instead, it functions purely as a feasibility constraint derived from tactile feedback. In our implementation, each classifier sample corresponds to a single synchronized control step containing the current tactile observation, the robot proprioceptive state, and the action evaluated at that step. The classifier therefore predicts local action feasibility for the present contact state rather than long-horizon task success. The classifier is trained on clean, normalized executed actions and queried within the same bounded normalized action space during sampling.

### 3.5 Phase awareness via single-sample scaling

Tactile relevance in manipulation is inherently phase-dependent: tactile feedback becomes critical during contact, while remaining largely uninformative in free space. Rather than explicitly modeling manipulation phases, we derive phase awareness directly from tactile feasibility.

Intuitively, when an action proposed by the vision policy is highly feasible under the current tactile observation, strong tactile guidance is unnecessary. Conversely, when feasibility is low, tactile constraints should exert greater influence on the sampling process. We therefore define the strength of tactile guidance as a function of action infeasibility.

At inference time, feasibility is evaluated on the current diffusion sample $a_{k}$ and the guidance scale is computed as:

$$\lambda \left(o_{t}^{h}\right)=\lambda_{\max}\left(1-C_{\phi }\left(o_{t}^{h},a_{k}\right)\right).$$ (6)

This single-sample formulation is sufficient in practice, as the diffusion process already introduces stochasticity across sampling steps. The resulting scalar $\lambda \left(o_{t}^{h}\right)$ directly modulates the strength of the tactile guidance, yielding implicit phase-aware control without explicit phase labels or additional networks. Together, Equations 3–6 define the forward diffusion process, feasibility estimate, classifier-guidance term, and phase-aware guidance scale used during sampling.

Algorithm 1 **DPTG: Diffusion Policy with Tactile Guidance (Inference).**

**Input:** Visual observation $o_{t}^{v}$ , tactile observation $o_{t}^{h}$ ; Vision diffusion policy $\epsilon_{\theta }$ ; Tactile feasibility classifier $C_{\phi }$ ; Noise schedule ${\left\{\eta_{k}\right\}}_{k=0}^{K}$ ; guidance scale $\lambda_{\max}$

**Output:** Action $a_{0}$

1 Sample initial noise $a_{K}∼N\left(0,I\right)$ ;
2 for k=K,…,0 do
  ;//Compute vision diffusion score
3 $\epsilon_{v}\leftarrow \epsilon_{\theta }\left(a_{k},o_{t}^{v},k\right)$ ;
 ;//Estimate phase-aware guidance scale
4 $\lambda \left(o_{t}^{h}\right)\leftarrow \lambda_{\max}\left(1-C_{\phi }\left(o_{t}^{h},a_{k}\right)\right)$ ;
 ;//Compute tactile feasibility gradient
5. $g_{h}\leftarrow \nabla_{a_{k}}\log C_{\phi }\left(o_{t}^{h},a_{k}\right)$ ;
 ;//Update diffusion sample
6. $a_{k-1}\leftarrow a_{k}-\eta_{k}\left(\epsilon_{v}+\lambda \left(o_{t}^{h}\right)\;g_{h}\right)$ ;
7 return $a_{0}$

### 3.6 Data collection and training

#### 3.6.1 Data collection

**FIGURE 3:**
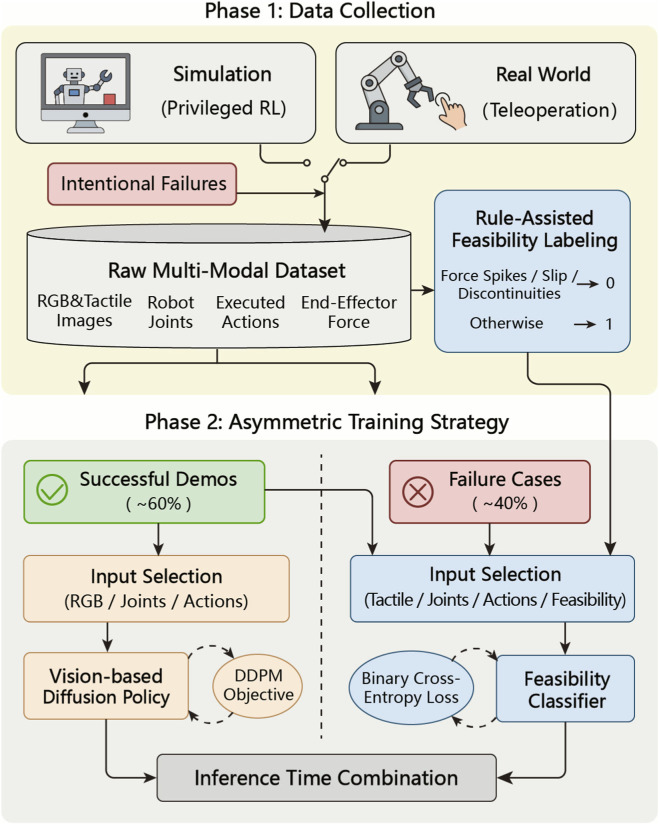
Data collection and training pipeline. Data are collected separately in simulation using a privileged RL policy and in the real world via teleoperation, including failure cases used to capture infeasible states. The vision-based policy (left) is trained exclusively on successful demonstrations to learn task distributions, whereas the feasibility classifier (right) utilizes the full dataset, including failure cases with rule-assisted feasibility labels, to learn physical constraints.

As shown in Figure 3, we collect multimodal trajectories in simulation and on the real robot, recording joint states, RGB observations, tactile images, end-effector force measurements, and executed actions. Simulation data are generated by a privileged RL policy with access to full simulator state, whereas real-world data are collected by human teleoperation with a SpaceMouse under diverse initial conditions. Tactile observations are depth images: in simulation, they come from a fingertip-view depth camera encoding object intrusion, while on the real robot they are reconstructed by Xense tactile sensors. Force observations are obtained from the simulated wrist force sensor in simulation and from end-effector force measurements on the real robot.

To train the feasibility classifier, we retain unsuccessful interactions, yielding approximately 40% failure cases. These failure segments supervise tactile feasibility learning but are excluded from training the action generation policy. In the real-world dataset, infeasible samples are mined from naturally occurring failed segments and exploratory contacts that later trigger the same safety rules used during post-processing.

#### 3.6.2 Rule-based label generation

Feasibility labels are assigned offline at the control-step level from synchronized force and tactile streams. Let $F_{t}$ denote the end-effector force vector at step t , $\Delta F_{t}=\|F_{t}-F_{t-1}{\|}_{2}$ the step-to-step force increment, $D_{t}=\|I_{t}-I_{t-1}{\|}_{1}/\left(HW\right)$ the normalized tactile-image difference, and $S_{t}$ the displacement of the tactile contact centroid between consecutive tactile frames. Before thresholding, force measurements are smoothed with a 3-step moving average and tactile images are normalized to [0,1].

A sample is labeled infeasible if any of the following conditions hold: $\|F_{t}{\|}_{2}>8.5\;N$ ; $\Delta F_{t}>2.5\;N$ ; $D_{t}>0.12$ and $S_{t}>3\;mm$ for two consecutive steps; or the sample lies within the last 10 control steps before a safety abort caused by hard collision, force escalation, or persistent jamming. All remaining samples are labeled feasible. Thus, positive labels correspond to contact responses inside the safety envelope of the current setup, whereas negative labels correspond to unsafe signatures or immediate pre-failure transitions.

These thresholds are fixed once for the sensing setup used in this paper and reused across all reported tasks and methods. They were selected from pilot trials by checking force spikes, tactile-depth changes, and contact-centroid motion before hard collisions or safety aborts, and then fixed across all tasks. They should therefore be interpreted as implementation-specific labeling parameters for the current force scale and tactile sensor mounting rather than as universal thresholds for other platforms.

#### 3.6.3 Training strategy

The vision-based diffusion policy and the tactile feasibility classifier are trained asymmetrically, reflecting their distinct roles in the proposed framework.

##### 3.6.3.1 Vision-based diffusion policy

The diffusion policy is trained using only successful demonstrations. It takes RGB observations and proprioception as input and predicts actions through conditional diffusion. Tactile and force signals are intentionally excluded. The policy is optimized using the standard DDPM objective in Equation 2, ensuring that action generation is driven by task-level visual cues rather than contact failures.

##### 3.6.3.2 Tactile feasibility classifier

The feasibility classifier $C_{\phi }\left(o_{t}^{h},a\right)$ is trained independently on a separate subset of the collected data. It takes tactile observations, robot joint states, and actions as inputs, and outputs a scalar feasibility score. The classifier is optimized using a binary cross-entropy loss. Simulation and real-world classifiers share the same architecture and labeling rules, but are trained on separate datasets.

The two components are combined only at inference time, and the complete inference procedure is summarized in Algorithm 1.

## 4 Experiments

We evaluate the proposed DPTG framework in both simulation and real-world settings to assess (i) the effectiveness of feasibility-centric diffusion control with implicit phase awareness, (ii) the resulting improvements in stability and safety for contact-rich manipulation, and (iii) the task-agnostic reuse of the tactile constraint module under a shared action space.

### 4.1 Experimental setup

#### 4.1.1 Simulation task

**FIGURE 4:**
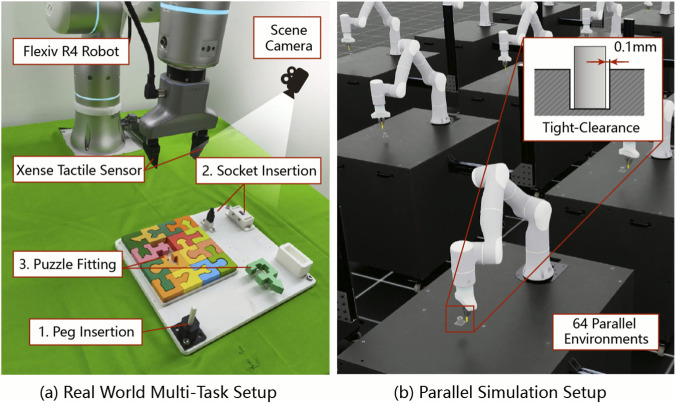
Experimental setup. **(a)** The real-world multi-task platform features a Flexiv R4 robot equipped with a scene camera and Xense tactile sensors, performing peg insertion, socket insertion, and puzzle fitting. **(b)** The parallel simulation environment utilizes 64 instances in NVIDIA Isaac Sim to facilitate large-scale data collection for a challenging peg-in-hole task with a tight 0.1 mm clearance.

As shown in Figure 4b, we implement the simulation environment using Isaac Sim NVIDIA (2025), leveraging 64 parallel scenes to accelerate both data collection and evaluation. The benchmark task is a canonical peg-in-hole insertion with a tight radial clearance of 0.1 mm (8.0 mm peg, 8.2 mm hole). This task involves distinct interaction phases, transitioning from free-space motion to constrained insertion. To strictly evaluate robustness, we randomize initial end-effector poses, hole offsets, and friction coefficients. These perturbations, combined with the tight tolerances, create challenging scenarios where visual feedback alone is insufficient, thereby necessitating precise tactile guidance to manage contact forces.

#### 4.1.2 Real-world tasks

As shown in Figure 4a, we further validate our approach on a real platform comprising a Flexiv robotic arm, end-effector-mounted Xense tactile sensors, and an RGB scene camera. Real-world experiments include peg insertion, plug-in socket insertion, and puzzle fitting, spanning tight-clearance alignment, asymmetric geometry, and repeated contact interactions; each trial starts from a randomized end-effector pose to reflect realistic initialization uncertainty and terminates upon success, a safety-triggered abort, or the preset rollout horizon.

### 4.2 Baselines and metrics

We compare DPTG against the following baselines:

- Vision-only DP: a unimodal diffusion policy conditioned solely on visual observations Chi et al. (2023).
- Feat-Concatenation: visual and tactile embeddings are concatenated at the feature level and decoded by a single diffusion policy.
- Symmetric Fusion: modality-specific diffusion policies combined symmetrically at the policy level using a product-of-experts formulation Chen et al. (2025).
- Ablations of DPTG: variants of the proposed method where tactile guidance is applied with constant strength, or modulated by a heuristic force threshold instead of the learned feasibility score.

We evaluate all methods using success rate and contact-related metrics, including peak contact force and force variance during contact. Failure modes on the real robot are also summarized and analyzed. Step count is reported for completeness but is not the primary focus. Real-world success rates are reported over n=50 trials per task.

### 4.3 Implementation details

Unless otherwise specified, all diffusion-based policies use the same visual backbone, denoising architecture, optimizer, and training schedule, and differ only in how tactile information is fused or whether tactile guidance is applied during inference. The visual observation is resized to 224×224 and encoded by a ResNet-18 backbone followed by global average pooling and a 256-dimensional projection head. Robot proprioception is processed by a two-layer MLP with hidden size 128 and concatenated with the visual embedding. The vision policy predicts short action chunks of length eight using a time-conditioned 1D U-Net denoiser with three resolution levels, base width 256, and sinusoidal diffusion-step embeddings. For tactile processing, the two tactile images are each resized to 128×128 and passed through a lightweight four-block CNN with channel widths 32-64-128-128, followed by global average pooling and a 128-dimensional projection, after which the two embeddings are concatenated and fused with the current robot state and action. The feasibility classifier is implemented as a three-layer MLP with hidden size 256 and a sigmoid output.

In all reported experiments, we use K=10 reverse diffusion steps. Runtime is measured on the deployment workstation equipped with an NVIDIA RTX 4090 GPU and an Intel Core i9-13900K CPU. End-to-end policy inference for DPTG requires approximately 70 m per policy update, corresponding to about 14 Hz high-level control updates. This 14 Hz rate refers only to the high-level policy thread. The policy fills an action buffer asynchronously; a consumer thread sends targets to the Flexiv robot at 30 Hz, while the 1 kHz impedance controller handles compliant servoing and safety limits. Baseline methods inherit these settings unless their modality integration mechanism explicitly differs.

### 4.4 Effectiveness of feasibility-centric control

We first evaluate whether feasibility-constrained sampling effectively leverages tactile feedback to improve task success compared to generative fusion strategies.

#### 4.4.1 Comparative performance

Quantitative results in both simulation (Table 1) and real-world experiments (Table 2) show that DPTG improves success rates over the evaluated baselines while producing more favorable contact-force statistics. In the challenging simulated peg-in-hole task, DPTG achieves an 87.5% success rate, significantly surpassing the symmetric policy fusion baseline at 75.7% and the vision-only baseline at 73.4% . On the real robot, DPTG yields improvements of 6– 16% across all three tasks.

**TABLE 1:** Main results in simulation (Mean ± std over 5,056 trials).

Task	Method	Success ↑ (%)	Steps ↓	Peak force ↓ (N)	Force var ↓ ( ${\text{N}}^{2}$ )
Peg-in-hole (tight)	Vision-only DP	73.4	45 ± 13	8.4 ± 3.2	5.6 ± 1.4
	Feat-concatenation	77.7	48 ± 10	6.9 ± 3.0	4.4 ± 1.2
	Symmetric fusion	75.7	46 ± 11	7.7 ± 3.1	5.0 ± 1.1
	DPTG (ours)	87.5	53 ± 10	3.6 ± 0.7	2.6 ± 0.6

Bold numerical values indicate the best result for each metric within each task.

**TABLE 2:** Main results on the real robot (Mean ± std over 50 trials per task).

Task	Method	Success ↑ (%)	Steps ↓	Peak force ↓ (N)	Force var ↓ ( ${\text{N}}^{2}$ )
Peg-in-hole (tight)	Vision-only DP	66	233 ± 58	10.1 ± 4.5	7.8 ± 2.1
	Feat-concatenation	68	241 ± 55	9.6 ± 4.1	7.0 ± 1.9
	Symmetric fusion	66	235 ± 62	11.4 ± 5.3	6.5 ± 1.8
	DPTG (ours)	74	255 ± 49	6.8 ± 3.2	3.9 ± 1.2
Plug-in-socket	Vision-only DP	48	254 ± 64	14.5 ± 5.2	8.6 ± 2.4
	Feat-concatenation	52	247 ± 61	13.1 ± 5.0	7.9 ± 2.2
	Symmetric fusion	50	260 ± 66	13.8 ± 5.1	7.4 ± 2.1
	DPTG (ours)	64	266 ± 56	6.2 ± 3.8	4.6 ± 1.4
Puzzle fitting	Vision-only DP	70	215 ± 70	9.8 ± 5.9	6.4 ± 2.7
	Feat-concatenation	72	235 ± 68	9.2 ± 5.6	6.7 ± 2.5
	Symmetric fusion	70	224 ± 72	9.9 ± 5.7	6.2 ± 2.4
	DPTG (ours)	78	259 ± 62	5.7 ± 3.5	4.1 ± 1.6

Bold numerical values indicate the best result for each metric within each task.

Notably, symmetric baselines provide only marginal gains over the vision-only policy. This result substantiates our core hypothesis that tactile feedback is fundamentally more effective as a corrective constraint than as a predictive action-generating modality. Since tactile signals are event-driven and temporally sparse, they lack the continuous density required for stable motion planning; forcing policies to directly generate actions from such signals often leads to degeneration or hallucination.

#### 4.4.2 Ablation study

**FIGURE 5:**
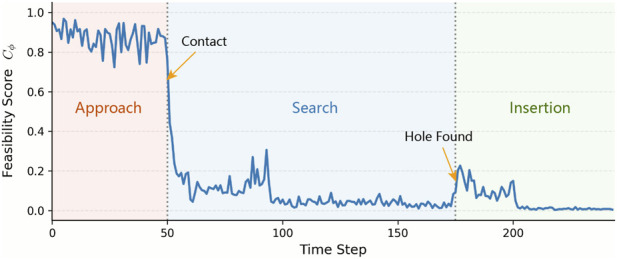
Evolution of the feasibility score $C_{\phi }$ during a real-world peg insertion. The curve reports the score of the denoised action selected at each policy update, with sharp drops marking higher-risk contact states, implicitly triggering stronger tactile guidance through phase-aware scaling.

To validate the contribution of the proposed feasibility-based scaling mechanism, we conduct an ablation study, with results summarized in Table 3. First, removing phase awareness and applying constant guidance (*w/o phase scaling*) causes the success rate to drop sharply from 87.5% to 70.3% . This degradation occurs because indiscriminate tactile gradients disturb free-space motion. As shown in Figure 5, DPTG mitigates this issue by deriving phase awareness from the learned feasibility score, which remains high in free space and decreases sharply upon contact onset. This adaptive scaling naturally concentrates tactile corrections in contact-rich phases while avoiding over-correction when contact constraints are inactive.

**TABLE 3:** Ablation studies in simulation (Peg-in-hole, tight).

Variant	Success ↑ (%)	Peak force ↓ (N)	Force var ↓ ( ${\text{N}}^{2}$ )
Full DPTG	87.5	3.6 ± 0.7	2.6 ± 0.6
Heuristic scaling	81.2	5.1 ± 2.8	3.9 ± 1.1
W/o phase scaling	70.3	3.8 ± 1.0	**2.4** ± 0.6
Vision-only	73.4	8.4 ± 3.2	5.6 ± 1.4

Bold numerical values indicate the best result for each metric.

Second, to verify the advantage of learned phase awareness over hand-crafted rules, we evaluate a “heuristic scaling” baseline where guidance is triggered only when the contact force exceeds a threshold of 2.0 N . While this heuristic outperforms constant guidance with an 81.2% success rate, it still lags behind the 87.5% achieved by full DPTG.

### 4.5 Robustness and interaction safety

A key advantage of modeling tactile feedback as a physical constraint is improved interaction safety, which we analyze through contact-force metrics and failure-mode shifts.

#### 4.5.1 Force regulation

**FIGURE 6:**
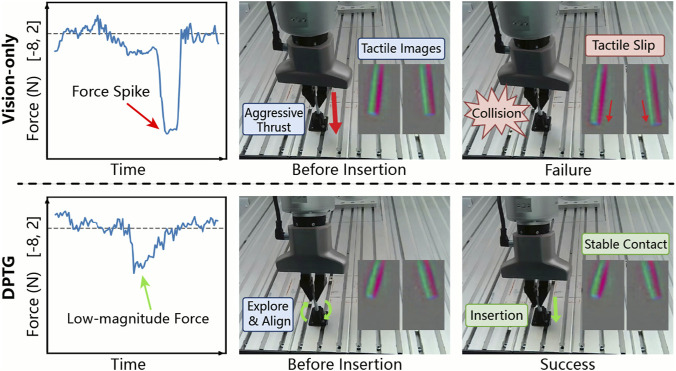
Comparison of contact interaction patterns. Top: vision-only diffusion policy executes aggressive insertion, causing force spikes and tactile slip that lead to collision and failure. Bottom: DPTG performs contact-aware probing and alignment with low-magnitude forces and stable contact, enabling successful insertion.

DPTG exhibits significantly safer and more consistent contact behaviors. In simulation, it reduces peak contact force by over 60% (8.4 N →3.6 N) compared to the vision-only policy. On the real robot, DPTG consistently achieves the lowest peak force and force variance across all tasks (Table 2), indicating improved safety and robustness under randomized initial configurations. These force metrics are consistent with the qualitative behaviors observed in Figure 6, where DPTG avoids the aggressive insertions and sharp force transients typically observed in vision-only policies.

On the other hand, DPTG may require slightly more control steps to complete a task. This trade-off reflects a deliberate behavioral shift: rather than forcing insertion under uncertainty, DPTG prioritizes contact-aware probing and fine alignment, leading to safer but more cautious interactions.

#### 4.5.2 Shift in failure modes

**FIGURE 7:**
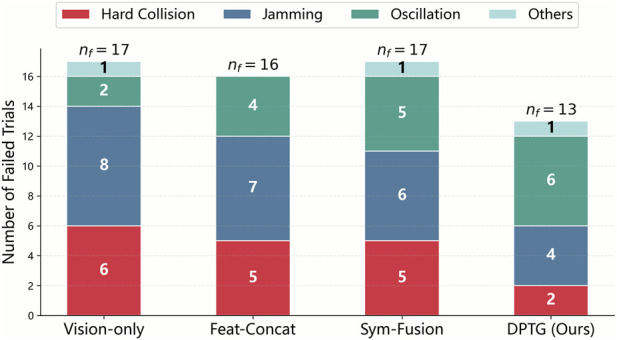
Failure mode breakdown on peg-in-hole (tight) on the real robot. The stacked bars represent the count of specific failure types. $n_{f}$ denotes the total number of failed trials out of 50.

The robustness improvement is further reflected in the qualitative distribution of failure modes. As shown in Figure 7, vision-only, feat-concatenation, and symmetric fusion baselines are dominated by *Hard Collisions* and *Jamming*, which are predominantly caused by aggressive actions during misalignment. In contrast, DPTG substantially suppresses these destructive failures, replacing them with *Oscillations*. These manifest as low-frequency iterative contact-retraction cycles: the policy cautiously probes the geometry, retracts upon detecting physical infeasibility or unexpected resistance, and re-attempts with local pose adjustments. In our evaluation, oscillation is counted as a failure if repeated advance-retract cycles exhaust the rollout horizon without net task progress. While this results in temporary stagnation without task progress, it prevents the excessive force accumulation and irreversible failures observed in baselines, reducing hardware risk.

This shift demonstrates improved robustness to visual ambiguity.

### 4.6 Task-agnostic reuse under a shared action space

We assess task-agnostic reuse by training the tactile feasibility classifier once on peg-in-hole data and then reusing it, without classifier retraining, for socket insertion and puzzle fitting. These target tasks share the same end-effector action space as the source task, although their object geometries and contact dynamics differ. Table 4 compares this setting against Vision-only and Symmetric Fusion, the latter trained with full task-specific tactile datasets.

**TABLE 4:** Cross-task reuse evaluation on the real robot (Mean ± std over 50 trials).

Task	Method	Tactile training data	Success ↑ (%)	Peak force ↓ (N)
Plug-in-socket	Vision-only DP	None	48	14.5 ± 5.2
	Symmetric fusion	Task-specific (socket)	50	13.8 ± 5.1
	DPTG (ours)	Peg-only (reused)	58	8.4 ± 4.0
Puzzle fitting	Vision-only DP	None	70	9.8 ± 5.9
	Symmetric fusion	Task-specific (puzzle)	70	9.9 ± 5.7
	DPTG (ours)	Peg-only (reused)	74	6.6 ± 3.5

The tactile feasibility classifier used by DPTG, is trained once on tight peg-in-hole interactions and reused for other tasks without retraining.

Bold numerical values indicate the best result for each metric within each task.

DPTG achieves the best overall trade-off in this reuse setting. The gain is larger for socket insertion, which is kinematically closer to peg insertion ( +10% over vision-only), and smaller for puzzle fitting (+4%) , while peak force on puzzle fitting decreases from 9.8 N to 6.6 N . These results support the modular design of DPTG and suggest that local feasibility constraints can be reused across related tasks sharing the same action space and tactile setup.

## 5 Conclusion and discussion

In this work, we introduced DPTG, a control framework that reformulates tactile sensing as a physical feasibility constraint and a phase-awareness regulator within a vision-driven diffusion policy. Across simulation and real-robot experiments, this asymmetric formulation improves task success while simultaneously reducing peak force and force variance. The cross-task results further suggest that a physics-grounded feasibility model can support task-agnostic reuse more readily than a task-specific tactile policy when the action space and tactile hardware remain fixed.

Despite its effectiveness, the proposed framework has limitations. First, tactile sensing in DPTG is limited to local feasibility evaluation and contact-aware modulation, so it does not directly support long-horizon planning. Second, the guidance assumes a continuous, differentiable action space. The present evaluation focuses on learning-based visual–tactile fusion under a fixed tactile setup, and the reuse experiments are limited to related insertion and fitting tasks sharing the same action space. Future work could extend the same feasibility-guided formulation to broader contact-rich settings, for example, sustained-contact manipulation, and make the classifier more contact-aware by conditioning it on the diffusion step k or injecting action noise during training.

## Data Availability

The raw data supporting the conclusions of this article will be made available by the authors, without undue reservation.
